# Use of hyaluronic acid associated with triamcinolone acetonide or ozone gas in the treatment of induced osteoarthritis in rabbits

**DOI:** 10.1590/ACB361201

**Published:** 2021-12-17

**Authors:** Giovanna Cristina Brombini, Sheila Canevese Rahal, Alexandre Todorovic Fabro, Ivan Felismino Charas dos Santos, Miriam Harumi Tsunemi, Jean Guilherme Fernandes Joaquim, Danuta Pulz Doiche, Jeana Pereira da Silva, Maria Jaqueline Mamprim

**Affiliations:** 1 MSc. Fellow PhD degree. Postgraduate Program in Animal Biotechnology - School of Veterinary Medicine and Animal Science (FMVZ) – Universidade Estadual Paulista (UNESP) - Botucatu (SP), Brazil.; 2 PhD. Department of Veterinary Surgery and Animal Reproduction (FMVZ) - Universidade Estadual Paulista (UNESP) - Botucatu (SP), Brazil.; 3 PhD. Department of Pathology and Legal Medicine – Universidade de São Paulo (USP) - Ribeirao Preto (SP), Brazil.; 4 PhD. Department of Veterinary Surgery and Animal Reproduction (FMVZ) - Universidade Estadual Paulista (UNESP) - Botucatu (SP), Brazil.; 5 PhD. Department of Biostatistics - Institute of Biosciences of Botucatu (IBB) - Universidade Estadual Paulista (UNESP) - Botucatu (SP), Brazil.; 6 PhD. Bioethicus Institute - Botucatu (SP), Brazil.; 7 PhD. Private Practice - Botucatu (SP), Brazil; 8 MSc, Fellow PhD degree. Postgraduate Program in Wild Animals - Department of Veterinary Surgery and Animal Reproduction (FMVZ) - Universidade Estadual Paulista (UNESP) - Botucatu (SP), Brazil.; 9 PhD. Department of Veterinary Surgery and Animal Reproduction (FMVZ) - Universidade Estadual Paulista (UNESP) - Botucatu (SP), Brazil.

**Keywords:** Polysaccharides, Adrenal Cortex Hormones, Ozone, Rabbits

## Abstract

**Purpose:**

To evaluate the effects of the intra-articular application of hyaluronic acid associated with triamcinolone acetonide, and ozone gas in the treatment of induced osteoarthritis in rabbit’s stifles.

**Methods:**

Twenty-one Norfolk rabbits were submitted to cranial cruciate ligament transection of the left stifle. After six weeks of the surgery, the rabbits were randomized assigned into three groups: G1 (control) – saline solution (0.9%); G2 – hyaluronic acid associated with triamcinolone; G3 – ozone gas, submitted to three intra-articular applications every seven days.

**Results:**

Significant differences occurred: osteophytes at medial femoral condyle (G2 > G1, G2 > G3) on radiography exam; thickening of the medial condyle (G1 > G3, G2 > G3) on ultrasound exam; osteophytes at lateral tibial condyle (G2 > G1, G2 > G3), and medial femoral condyle (G1 > G2, G3 > G1) on computed tomography. Histologically, mean values of chondrocytes in the femur and tibia in G3 and G2 were statistically lower.

**Conclusions:**

The intra-articular injection of hyaluronic acid associated with triamcinolone accentuated degenerative joint disease by imaging and macroscopic evaluations, and by histological findings, this treatment and the ozone gas treatment showed similar effects and were inferior to the saline solution (0.9%).

## Introduction

Osteoarthritis is a multifactorial disorder characterized by gradual cartilage loss, joint inflammation, osteophyte formation, and subchondral bone remodeling that may induce movement limitation, pain, lameness, and loss of function^1^^-^ 3. Since there is no definitive cure for osteoarthritis, treatment goals include pain relief and maintenance/or improvement of joint mobility, aiming to limit functional impairment^3^^-^ 5. In addition, efforts should be made to reduce the damage or improve the articular cartilage, and to restore the physiological conditions of the joint^4^^,^ 6. The treatments include surgical procedures or conservative approaches, such as reduction of modifiable risk factors, intra-articular therapies, physical modalities, as well as systemic pharmacological treatments^3^^,^ 6. Among substances used intra-articularly for the osteoarthritis treatment, corticosteroids, hyaluronic acid or combinations, and blood-derived products may be cited 1 ^,^ 7.

Corticosteroids act in osteoarthritis through anti-inflammatory actions and immunosuppressive effects, but the mechanism of action is complex^1^. Intra-articular corticosteroid injections can decrease the synthesis of inflammatory mediators, such as interleukin 1 beta (IL-1β), tumor necrosis factor alpha (TNFα), and cyclo-oxygenase enzyme 2 (COX-2) in synovial fluid^8^. However, according to the literature^1^^,^ 3, corticosteroids should be considered an adjunct to moderate to severe pain treatment because of the short-term pain relief, and, on the other hand, corticosteroids can accelerate cartilage damage^3^.

The use of intra-articular exogenous hyaluronic acid is based on the modification of effects of clinical signs^4^^,^ 9. Pain relief mechanisms include proteoglycan and/or glycosaminoglycan synthesis, anti-inflammatory effect, and maintenance of viscoelasticity^10^. Furthermore, reduction of pro-inflammatory cytokines, free radicals and proteolytic enzymes in the synovial fluid seems to provide cartilage protection^11^. Thus, the effectiveness of the exogenous hyaluronic acid is associated with intra-articular lubrication and anti-inflammatory, analgesic, and chondroprotective effects^10^. In turn, the intra-articular application of a combination of corticosteroids with hyaluronic acid, or even administration of both products separated, has been performed to enhance pain relief, being short-term effect because of corticosteroid and long-term effect and cartilage protection due to action of hyaluronic acid^12^^-^ 15.

An option less frequently used to intra-articular treatment of osteoarthritis is the ozone gas, that has been associated with fast pain relief, the disappearance of edema, decongestion, increased mobility, and delay in the degenerative process^16^^,^ 17. Ozone gas injected into synovial fluid may be responsible for the generation of reactive oxygen species (ROS) and lipid oxidation products (LOPs) that act by inactivating or inhibiting pro-inflammatory cytokines and proteolytic enzymes, as well as stimulating the production of chondrocytes and fibroblasts, favoring matrix synthesis^5^^,^ 17. Thus, ozone infiltration seems to have the abilities to accelerate anabolism and to promote an improvement in vascularization of cartilage and bone^17^.

Therefore, this study aimed to evaluate the effects of the intra-articular application of the cross-linked hyaluronic acid associated with triamcinolone acetonide, and ozone gas in the treatment of induced osteoarthritis in rabbit’s stifles.

## Methods

This study followed the guidelines for the care and use of laboratory animals and was approved by the Institutional Ethics Committee for the Use of Animals (CEUA), no. 0011/2017.

### Animals and experimental design

Twenty-one healthy male Norfolk rabbits, approximately five months of age and mean body mass of 4 kg, were used. The rabbits were housed at the Experimental Research Unit (UNIPEX) of Botucatu Medical School in individual cages (length = 50 cm, width = 60 cm, height = 60 cm) and received a commercial pelleted diet and water *ad libitum*.

All rabbits were submitted to the cranial cruciate ligament transection of the left stifle joint for osteoarthritis induction, based on a study previously described^18^. The right stifle of each group was determined as control group (unoperated), for macroscopic and histopathological analyses.

### Anesthesia and surgical procedure

Anesthesia was performed with a combination of ketamine chloridrate (50 mg/kg) and xylazine (1 mg/kg) administered in rectus femoris muscle of the right hind limb. The rabbits were placed in dorsal recumbence, and, after aseptic skin preparation, a medial parapatellar arthrotomy was done in the left hind limb. After cranial cruciate ligament transection, the patella was returned to the femoral trochlea, and the articular capsule was closed with a simple continuous suture using poliglactine (3-0). The subcutaneous layer was closed with a simple continuous pattern (poliglactine 3-0) and skin with a simple interrupted pattern (polypropylene 3-0). Ceftriaxone (50 mg/kg) was administered intramuscularly immediately before the surgery and 12 hours after it, and tramadol hydrochloride (0.02 mg/kg, 8 h, subcutaneous) for three days. The skin sutures were removed after 10 postoperative days.

### Treatments

After six postoperative weeks, the rabbits were randomly assigned into three groups, according to intra-articular treatment:

• Group 1 (control): 0.3 mL of saline solution (0.9%);

• Group 2: 0.3 mL of hyaluronic acid associated with triamcinolone;

• Group 3: 30 μg/mL of ozone gas.

In each group, three intra-articular applications were administered every seven days.

### Imaging evaluations

The stifle joints were evaluated by imaging studies (radiography, ultrasound and computed tomography), performed at following time-points: before (TP0) and six weeks after cranial cruciate ligament transection (TP1), and five weeks after last intra-articular application (TP2). All imaging evaluations were done by examiners experienced in diagnostic imaging blinded to the treatment.

Mediolateral and extended caudocranial views were taken with a digital radiography system GE®, with 90-cm focal-film distance and settings of 46 kV and 200 mA. Radiographic changes were evaluated in scores, based on a study previously described^19^. The right and left stifle joints were evaluated as follows: joint space width (0 = normal, 1 = slightly reduced, 2 = moderately reduced, 3 = severely reduced); formation of osteophytes at tibial condyles (medial and lateral) and/or femoral condyles (medial and lateral) (0 = absent, 1 = mild, 2 = moderate, 3 = severe), at fabella (medial and lateral) and/or sesamoid of the popliteal muscle (0 = absent, 1 = mild, 2 = moderate, 3 = severe); and sclerosis of the subchondral bone (present or absent).

The ultrasonographic evaluation of the stifle joints was performed with the rabbits in dorsal recumbence. An ultrasound system GE® using a 10 MHz linear transducer was used. Images were obtained in sagittal and transversal planes. Joint effusion, capsular thickening, and thickness of the medial and lateral femoral condyle were evaluated.

Computed tomography was carried out using a spiral scanner Shimadzu®, with the rabbits in ventral recumbence and hind limbs extended. Transverse cross-sections of 1-mm thickness were made from the distal third of the femur to the proximal tibia, with a protocol of 120 kVp and 100 mA and rotation time of 1 second. The images were reconstructed using dedicated software 3D Voxar®, in dorsal and sagittal planes. Joint space width (0 = normal, 1 = slightly reduced, 2 = moderately reduced, 3 = severely reduced); formation of osteophytes at femoral condyle (medial and lateral) and/or tibial condyle (medial and lateral) (0 = absent, 1 = mild, 2 = moderate, 3 = severe), and at fabella (medial and lateral) and/or popliteal sesamoid (0 = absent, 1 = mild, 2 = moderate, 3 = severe) were analyzed.

### Macroscopic and histological analyses

All animals were euthanized for macroscopic and histological evaluations of the stifle joints five weeks after the last intra-articular application (TP2). After the anesthesia using the same protocol previously mentioned, the rabbits received sodium thiopental (5mg/kg) and potassium chloride (10 mg/kg) intravenously until cardiorespiratory arrest. The stifle joints were evaluated macroscopically for signs of degenerative changes, and scored by absent or present.

The stifles were harvest and fixed 10% buffered formalin for 48 h, followed by storage in 70% alcohol and decalcification with 10% nitric acid. After decalcification, the medial femoral and tibial condyles were sectioned longitudinally in a block that was embedded in paraffin. Then, 3-μm sagittal sections were cut and stained with hematoxylin and eosin (H&E) and picrosirius red.

H&E staining allowed morphometric analysis of chondrocytes, and the following qualitative assessments (present or absent):

• Zone I: regularity of the joint surface, parallel organization of chondrocytes, the flattened appearance of chondrocytes, cleft or fissures, and chondrocyte niches;

• Zone II: distribution of chondrocytes, cleft or fissures, and chondrocyte niches, and vascular invasion;

• Zone III: Identification of the tidemark line, cleft or fissures and chondrocyte niches, vascular invasion;

• Zone IV: distribution of chondrocytes, cleft or fissures and chondrocyte niches, integrity of this area to the subchondral bone and vascular invasion.

Picrosirius red staining was used to stain collagen fibers. The images were captured through a digital camera on the led microscope by polarized light and analyzed using Image-Pro Plus 7 software. The density of collagen was measured in the samples in ten randomly selected microscopic fields at magnification of ×200. The threshold for collagen fibers was established for all slides after the contrast was enhanced to the point at which the fibers were easily identified as green or orange bands. The density of the collagen and elastic fibers was expressed as the ratio between the measured fibers divided by the total area studied ×100.

### Statistical analysis

After data normality was tested using the Shapiro-Wilk test, Kruskal-Wallis and chi-square tests were used to evaluate alterations observed in the imaging studies and gross exam.

The counting of chondrocytes and collagen fibers in the right stifle joint of the three groups did not show significant differences. Thus, all data were grouped as a control group (unoperated). The data were evaluated by the t-test. Statistical analysis was performed at Statistical Package for Social Sciences (SPSS) v. 13 software (SPSS, Inc., Chicago, IL, 2004). Differences were considered statistically significant at p ≤ 0.05.

## Results

### Imaging evaluations

Radiographic examination showed statistically differences, as follows: formation of osteophytes at medial femoral condyle in TP2 (G2 > G1, G2 > G3) (P = 0.0216), and at medial tibial condyle in TP1 (G3 > G1; G3 > G2) (P = 0.0216). Ultrasound evaluation found significant differences in the thickening of the medial condyle in TP2 (G1 > G3; G2 > G3) (P = 0.0059). Joint effusion and capsular thickening have been identified particularly in the cranial compartment. Computed tomography (CT) showed significant differences, as follows: formation of osteophytes at lateral tibial condyle in TP1 (G3 > G1, G3 > G2) (p = 0.0408) and TP2 (G2 > G1, G2 > G3) (P = 0.0408), and at medial femoral condyle in TP2 (G1 > G2 and G3 > G1) (P = 0.0216).

**Figure 1:**
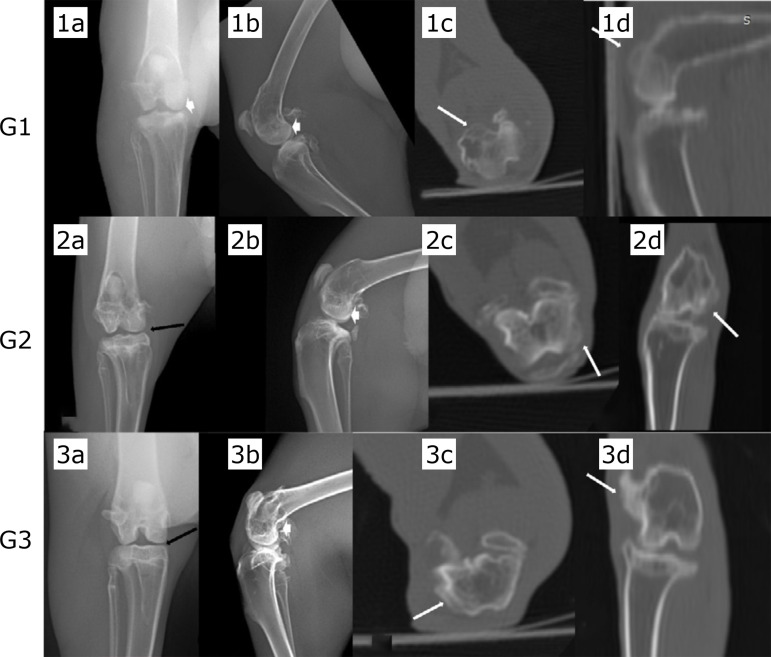
Caudocranial **(1a,2a,3a)** and mediolateral **(1b,2b,3b)** radiographic views, and computed tomographic images in transverse **(1c,2c,3c)**, sagittal **(1d)**, and dorsal **(2d,3d)** planes of the left stifle, in rabbits treated with saline solution **(G1)**, hyaluronic acid associated with triamcinolone **(G2)** and ozone gas **(G3)**. Observe sclerosis of the subchondral bone (*white arrowheads*), the formation of osteophytes at femoral condyle (*white arrow*), and reduced joint space width (*black arrows*).

**Figure 2:**
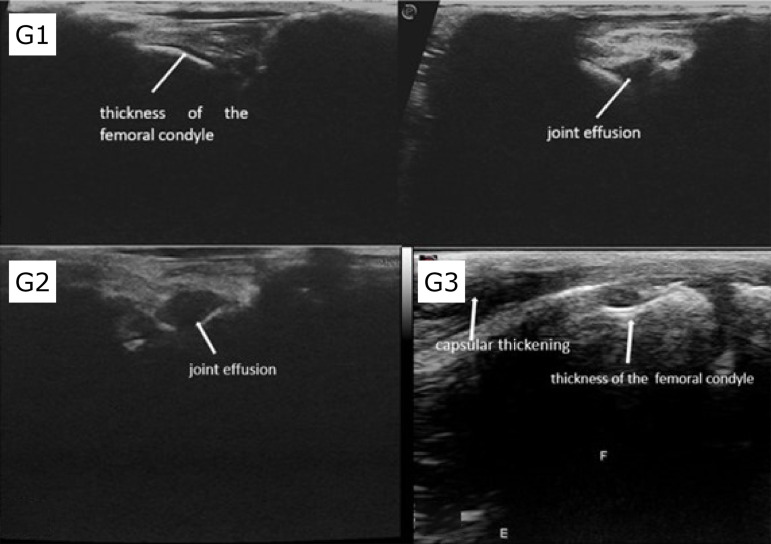
Ultrasound images of the left stifle on the longitudinal plane, in rabbits treated with saline solution **(G1)**, hyaluronic acid associated with triamcinolone **(G2)**, and ozone gas **(G3)**. Joint effusion, capsular thickening, and thickness of the medial and lateral femoral condyle were noticed.

Figures 1 and 2 show the findings seen in the groups, respectively, in radiographs and CT, and ultrasound.

### Macroscopic and histological analyses

All treated stifles showed signs of osteoarthritis with irregularity in the articular surface of the patella and trochlea, and osteophytes at the medial and lateral femoral condyles, which did not differ statistically among groups. The increase in joint volume (P = 0.0216) and thickening of the joint capsule (P = 0.02098) were more intense in G2 than in G1 and G3. The stifles in the control group (unoperated) showed slight alterations.

**Figure 3:**
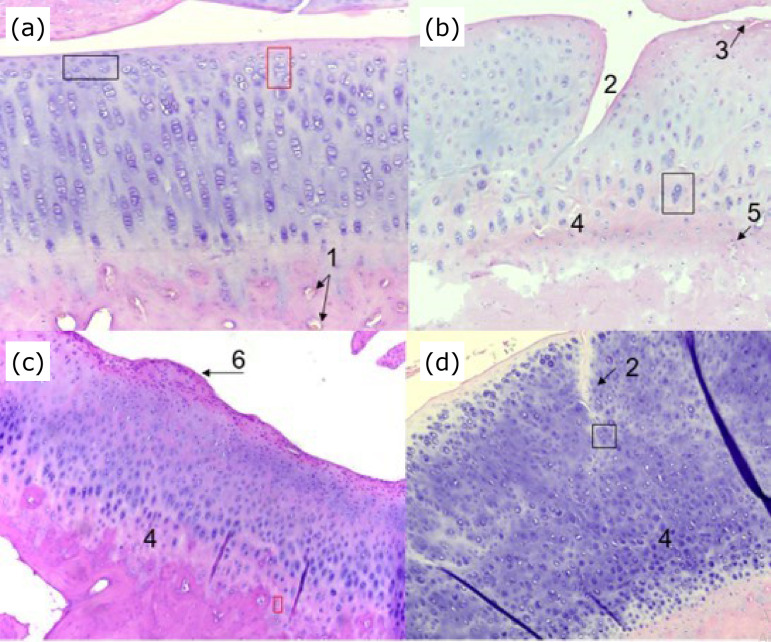
Condylar surface of the rabbits’ femur stained with H&E (10X) at five weeks after last intra-articular application. **(a)** Unoperated: chondrocytes with an elliptic shape in articular surface (*black rectangle*); column of chondrocytes in zone II (*red rectangle*); 1 = vascularization. **(b)** Saline solution (0.9%) treatment (control): chondrocyte disarrangement in all zones, loss of chondrocyte column in Zone III; 2 = crack extending from the surface to transition-zone; 3 = desquamation of the cartilage surface; 4b = poor definition of the tidemark; 5 = irregular border between cartilage and bone tissue; chondrocyte niches (*square*). **(c)** Hyaluronic acid with triamcinolone treatment: 4c = poor definition of tidemark and chondrocyte clones (*red square*), 6 = osteophyte formation. **(d)** Ozone gas treatment: 2 = crack extending from the surface to transition-zone; 4d = total loss of tidemark; degenerated chondrocyte (*square*).

Histological analysis of H&E-stained tissue sections revealed that no group had cartilage necrosis, but intense chondrocyte disorganization was detected in all treated groups and in all zones (Fig. 3). No statistically significant difference was identified regarding regularity, organization, and preservation of the flattened shape of the chondrocytes in G2 and G3 when compared to G1 in zone I. Furthermore, tidemark and chondrocyte columns were hard to visualize in G3. In zone IV (calcified), the presence of cracks was statistically increased in G1 (P = 0.026) than in G2 and G3. The other qualitative variables assessed as present or absent did not differ among groups. The mean of chondrocytes in both femoral and tibial condyles in G2 and G3 were statistically lower than in the control group (unoperated). No difference occurred between G1 and the control group (unoperated). The mean of chondrocytes including all treated stifles (G1, G2, and G3) was lower in both femoral and tibial condyles compared to the control group (unoperated).

**Figure 4:**
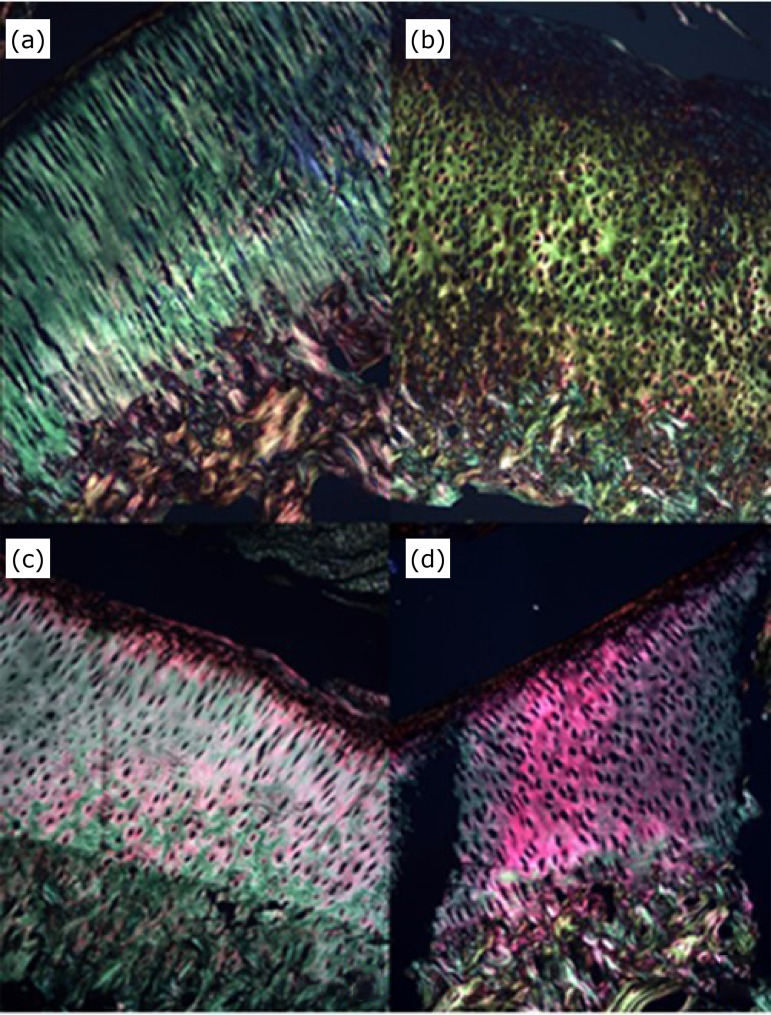
Condylar surface of the rabbits’ femur stained with picrosirius red (x10) at five weeks after last intra-articular application showing reddish-stained collagen fibers. **(a)** Unoperated, and treated with **(b)** saline solution (0.9%), **(c)** hyaluronic acid associated with triamcinolone, and **(d)** ozone gas.

Histological sections stained with picrosirius red demonstrated loss in the alignment of the collagen fibers in all treated groups (Fig. 4). The superficial portion of the cartilage had dark color (less collagen), and the intermediate zone had a more reddish color (more collagen). The proportion of collagen fibers including femoral and tibial condyles was statistically higher in G2 and G3 than in the control group (unoperated). No difference occurred between G1 and the control group (unoperated).

## Discussion

The study evaluated two treatment modalities in osteoarthritis induced by transection of the cranial cruciate ligament using rabbits as experimental models and identified that the degenerative process remained intense despite these treatments.

The three imaging exams allowed the detection of osteoarthritis six weeks after the transection of the cranial cruciate, and some differences were observed among groups. Comparing the post-treatment groups, G2 had higher scores compared to G3 and G1 regarding osteophytes in the medial femoral condyle and lateral tibial condyle, respectively, according to radiographic and CT scans. G2 also showed greater thickening of the medial condyle on ultrasound compared to G3. Thus, the imaging findings suggested more intense osteoarthritis in the combination of reticulated hyaluronic acid with triamcinolone. In addition, macroscopically, the variables joint volume and capsular thickening were greater in G2 compared to G1 and G3. Studies in human patients that received intra-articular hyaluronic acid and corticosteroids interspersed or in association showed significant improvement in pain relief and functional recovery compared to only hyaluronic acid^13^^-^ 15. Both parameters could not be evaluated in the present study because pain assessment in rabbits is not standardized and classified for joint procedures, and visual gait analysis did not allow to differentiate lameness degrees due to the severity of the injury.

Histological analysis also showed significant decrease in chondrocytes at femur and tibia in the G2 compared to the control group (unoperated), but there were no differences with G1 and G3. However, G2 showed a trend in the regularity, better organization, and preservation of the flattened shape of the chondrocytes when compared to G1. On the opposite, stifle osteoarthritis induced by collagenase injection in rabbits treated intra-articularly with cross-linked hyaluronic acid, or a mixture of cross-linked hyaluronic acid, 1% ropivacaine hydrochloride, and triamcinolone acetonide showed improvement of the articular cartilage degeneration (grade II of osteoarthritis) compared to the saline solution (0.9%) group (grade III of osteoarthritis) on histologic analysis^20^. In turn, osteoarthritis treated 30 days after transection of the cranial cruciate ligament in rabbits was detected with higher healing rate and decreased degeneration in the group injected with hyaluronic acid and cortisone by intercalated form, compared to groups injected only with hyaluronan or cortisone^12^.

On the other hand, picrosirius red staining showed a statistically higher proportion of collagen fibers in G2 than in the control group (unoperated) that may be related to the intensity of osteoarthritis. Collagen is one of the major components of the extracellular matrix, whose fibers resist tensile forces and keep the proteoglycans within the joint^21^. The synthesis of type II collagen is increased in osteoarthritis to maintain the survival and integrity of the cartilage^22^.

Several types of corticosteroids and hyaluronic acid have been used in rabbits’ studies^12^^,^ 20 ^,^ 23 ^,^ 24. The corticosteroid incorporated into the product was triamcinolone, that is one of the corticosteroids of intra-articular injection used in human patients^1^^,^ 7 ^,^ 25. In a systematic review, triamcinolone appeared to be more effective than methylprednisolone and betamethasone, but with pain relief for only one week, which suggested the need for other treatment modalities for chronic pain^26^. Furthermore, the use for prolonged periods and at high doses can promote side effects, such as damage to the cartilage and knee bursa, causing worsening of osteoarthritis and, consequently, more pain^3^^,^ 7. Therefore, further studies with different proportions between corticosteroids and hyaluronic acid must be carried out.

Regarding hyaluronic acid, the one used in the current study was cross-linked and had molecular weight of 4.2 Da. Products with high molecular weight containing cross-linked molecules have been produced to obtain greater elastoviscosity and intra-articular longer time^4^. The hyaluronic acid produced from rooster combs (avian type) has a higher risk of immunogenicity than those ones produced by recombinant technology (biofermentation) that maintains purity and without allergenic potential^4^^,^ 10.

The intra-articular dose of ozone gas used in the current study was 30 μg/mL in 0.3 mL (corresponding to 9 μg per joint), with three applications every seven days. In human patients, there are several variations regarding the concentration and volume of intra-articular ozone and the number of sessions^27^^,^ 28.

The ozone gas has been used periarticular, intra-articular, and subcutaneous in the knee^5^^,^ 17. The oxygen-ozone concentration of 10 μg/mL has been recommended, with a gas volume of 20 mL for intra-articular injections and 10 mL for periarticular and subcutaneous injections^5^; therefore, inferior to that was used in the present study. However, there are studies in human patients that the doses of 20^29^^,^ 30 and 30 μg/mL^31^^,^ 32 were used. Furthermore, a study carried out in rats with an intra-articular infusion of 0.05 mL at the concentration of 20 μg of ozone/mL of oxygen (three times a week for three weeks) showed no changes in mobility or histological damage to the articular cartilage or synovial membrane, or even systemic or local undesirable effects^33^.

The histologic analysis of the G3, like G2, showed decrease in chondrocytes of the femur and tibia compared to the control group (unoperated), but with a tendency towards regularity, organization, and preservation of chondrocytes compared to G1. In addition, no cracks were detected in zone IV, unlike the other groups. On the other hand, G3, similarly to G2, showed a statistically higher proportion of collagen fibers than the control group (unoperated). Several studies in human patients that evaluated the positive effects of intra-articular administration of ozone gas have been based on the improvement of pain, stiffness, and joint function, as well as the quality of life^27^^,^ 29 ^-^ 31 ^,^ 34 ^,^ 35 mainly in short or medium term^36^. As mentioned before, these parameters could not be evaluated in the present study. Thus, studies with less severe osteoarthritis and gait analysis must be performed. In addition, limitations of the study could be considered, as the lack of a corticosteroid group, a cross-linked hyaluronic acid group, and an ozone and triamcinolone group.

## Conclusion

The intra-articular injection of hyaluronic acid associated with triamcinolone accentuated the degenerative joint disease by the imaging and macroscopic evaluations, and by the histological findings this treatment and the ozone showed effect similar and inferior to saline solution (0.9%).
